# Microneedle patches containing mesoporous polydopamine nanoparticles loaded with triamcinolone acetonide for the treatment of oral mucositis

**DOI:** 10.3389/fbioe.2023.1203709

**Published:** 2023-05-05

**Authors:** Xiaoying Qu, Xiaoli Guo, Tingting Zhu, Zhe Zhang, Wanchun Wang, Yuanping Hao

**Affiliations:** ^1^ Department of Stomatology, School of Stomatology of Weifang Medical University, Weifang, China; ^2^ School of Stomatology of Qingdao University, Qingdao, China; ^3^ Qingdao Stomatological Hospital, Qingdao, China

**Keywords:** oral mucositis, dissolving microneedle, mesoporous polydopamine nanoparticles, triamcinolone acetonide, oral ulcer

## Abstract

Oral mucositis (OM) is the most common disease of the oral mucosa, which affects people’s daily production and life. Triamcinolone ointment is the common clinical drug for OM treatment. However, the hydrophobic properties of triamcinolone acetonide (TA) and the complex microenvironment of the oral cavity led to its low bioavailability and unstable therapeutic effects on ulcer wounds. Herein, dissolving microneedle patches (MNs) composed of mesoporous polydopamine nanoparticles (MPDA) loaded with TA (TA@MPDA), sodium hyaluronic acid (HA), and *Bletilla striata* polysaccharide (BSP) are prepared as the transmucosal delivery system. The prepared TA@MPDA-HA/BSP MNs exhibit well-arranged microarrays, high mechanical strength and fast solubility (<3 min) properties. In addition, the hybrid structure improves the biocompatibility of TA@MPDA and expedites oral ulcer healing in the SD rat model through the synergistic anti-inflammatory and pro-healing effects of microneedle ingredients (hormones, MPDA and Chinese herbs extracts), with 90% less amount of TA compared with Ning Zhi Zhu^®^. TA@MPDA-HA/BSP MNs are shown to be their great potential as novel ulcer dressings for OM management.

## 1 Introduction

Oral mucositis (OM) is an inflammatory disease caused by necrosis and shedding of oral mucosal tissue, which can involve the epithelial layer and connective tissue (An et al., 2022). At the stage of the disease’s progression, the mucosa will be congested, erosive, prolonged, and may even become oral cancer. Characterized by severe pain, OM affects the patient’s eating and speaking functions and reduces the quality of life. OM is closely associated with vitamin or trace element deficiency, genetic and immune factors, local trauma, and allergenic infections, and other systemic diseases such as head and neck chemotherapy (Bilodeau and Lalla, 2019; Elad et al., 2022). These processes lead to cell and microvascular injury, the release and amplification of inflammatory cytokines, the generation of reactive oxygen species (ROS), and the destruction of mucosal epithelial integrity. Tumor necrosis factor-α (TNF-α) plays an important role in the pathogenesis of autoimmune diseases. In response to the inflammatory response of immune cells, TNF-a is released on a large scale, resulting in oral epithelial damage and eventually the formation of ulcerated surfaces. Platelet endothelial cell adhesion molecule-1 (CD31), a member of the immunoglobulin superfamily, plays an important role in clearing aging neutrophils and is closely related to angiogenesis from the body. During the healing stage, epithelial hyperplasia causes the mucosa to gradually heal. Therefore, inhibiting inflammatory response, clearing ROS and promoting cell proliferation are the key to the treatment of oral mucositis. Triamcinolone acetonide (TA) is commonly used in the treatment of OM as an adrenal glucocorticoid, which has highly effective anti-inflammatory, immunosuppressive and anti-allergic effects (Padula et al., 2018; Seon-Woo et al., 2019). However, TA is currently only produced as a topical formulation (ointment and gel) with poor water solubility and minimal oral bioavailability. Moreover, the oral cavity is a dynamic and moist environment where it is difficult for ointment and gel to exert effects in the lesion area due to saliva flushing, involuntary oral movements and swallowing (Zheng et al., 2021).

Recently, microneedles (MNs), as a highly effective transdermal drug delivery form that can break through the mucosal barrier, have attracted extensive attention in the treatment of oral diseases (Moore et al., 2022). MNs can be classified in a variety of ways. The most common way is to divide microneedles into solid microneedles, coated microneedles, hollow microneedles, soluble microneedles and hydrogel microneedles. Among them, Hydrogel microneedles (HMNs) are prepared from expandable crosslinked polymers. In a transdermal drug delivery system, drugs enter the body through loose apertures to achieve transdermal drug delivery. Compared with other kinds of microneedles, hydrogel microneedles can increase the drug loading of microneedles by setting up a reservoir. However, there are also some problems such as large pores left after the removal of loose hydrogel, long recovery time, and easy infection. Dissolving MNs composed of hydrophilic polymers have become popular biological drugs because of their simple manufacturing process, good biocompatibility, and precise transdermal drug delivery (Bian et al., 2021; Jing et al., 2021; Sartawi et al., 2022). With certain mechanical strength, dissolving MNs can insert into the oral mucosa, form multiple tiny channels, and subsequently dissolve into the liquid to release drug quickly. Therefore, MNs has become one of the most desirable alternatives to traditional local drug for the delivery of small molecules, large molecules and nanoparticles (Hao et al., 2020; Qin et al., 2020; Vora et al., 2020). *Bletilla striata* polysaccharide (BSP) is a kind of natural glucomannan with high biosafety and low price. BSP has anti-inflammatory, pro-coagulant, antibacterial and antioxidant activities, thus promoting wound healing (Wu et al., 2021; Zhang et al., 2022). Hu et al. first prepared MN using BSP in 2018 (Hu et., 2018). The results showed that MNs consisting of 24% BSP could penetrate the skin of rats. Sodium hyaluronic acid (HA) is a substance extracted from cockscomb and can also be prepared by lactic acid fermentation. HA with good biocompatibility has been found extensively used for tissue regeneration (Hemshekhar et al., 2016; Agarwal et al., 2020; Xing et al., 2020). HA in the form of microneedles has been applied in drug transdermal delivery systems for wound dressings and superficial tumor treatment (Hao et al., 2020; Peng et al., 2021; Saha and Rai, 2021). We have recently developed hyaluronic acid microneedle patch to improve the delivery of soluble small-molecule drugs into rat oral mucosa, and to protect the adhesive layer from mouth movement and saliva (Zhu et al., 2022). In this study, we tried to combine HA and BSP to enhance the therapeutic effect. Low molecular weight HA can enhance the mechanical strength of microneedle tip, and cooperate with BSP to give full play to anti-inflammatory and wound healing effects.

So far, many materials have been used as carriers to synthesize composite materials, such as montmorillonite (Li et al., 2022; Zhu et al., 2022), polydopamine (PDA) and so on. PDA is a new nanoscale material that has attracted more attention because of its good biocompatibility, near-infrared absorption, scavenging ability of oxidative free radicals, good antimicrobial properties, and excellent drug carrier (Bao et al., 2018; Wang et al., 2018). Mesoporous polydopamine nanoparticles (MPDA NPs) have a significantly higher drug-loading capacity than conventional PDA. The abundant functional groups and mesoporous structure on its surface enable it to combine with drug molecules through chemical bonding, electrostatic adsorption, π-π stacking and spatial structure (Tang et al., 2015; Zhu et al., 2021).

**FIGURE 1:**
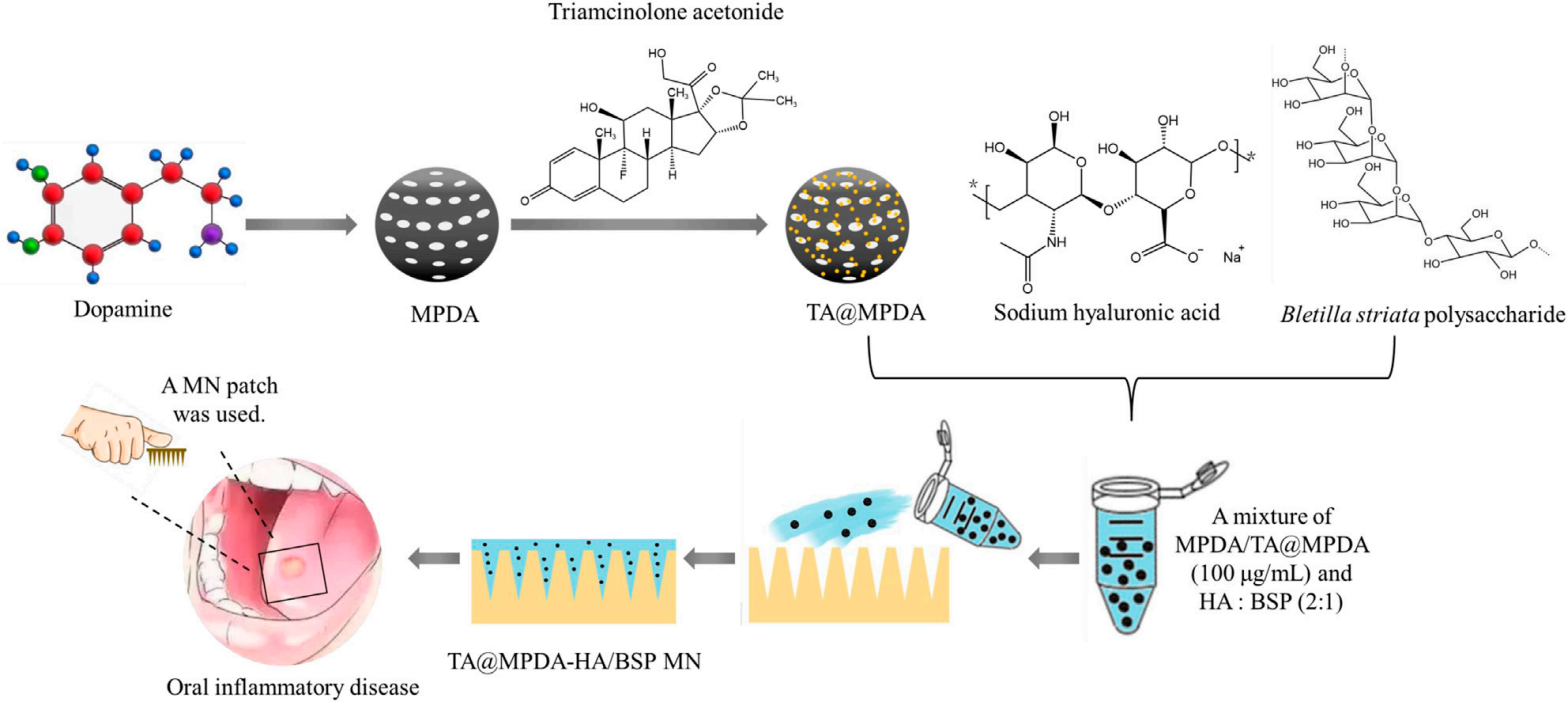
Schematic illustrations of the preparation of TA@MPDA-HA/BSP MN patch and its application in oral inflammatory disease.

Here, we may reasonably hypothesize that i) MPDA NPs may be the ideal carrier for TA, both to improve the bioavailability of TA and to exert the anti-inflammatory effects of MPDA NPs; ii) the combination of TA@MPDA NPs with dissolving HA/BSP microneedle may produce synergistic effects in the treatment of OM, reducing the dosage of the hormone administered. To test our hypotheses, TA@MPDA NPs were prepared through π-π stacking and encapsulated in the HA/BSP dissolving microneedle patch *via* a one-casting fabrication process for oral mucositis treatment. The schematic illustration of the TA@MPDA-HA/BSP MN patch is shown in Figure 1. The sharp tips of microneedles penetrated the mucosal barrier and delivered the drug deep without causing adverse reactions. TA could be sustainably released at the lesion, synergistic with MPDA to inhibit ROS-induced inflammation reaction. At the same time, the combination of TA@MPDA and HA/BSP could significantly increase the human oral keratinocytes (HOK) and primary human gingival fibroblasts (hGFs) proliferation and migration. The TA@MPDA-HA/BSP MN patch exhibited excellent treatment effects for accelerating oral ulcer closure. Taken together, the constructed microneedle patch is a promising candidate for oral inflammatory disease.

## 2 Materials and methods

### 2.1 Materials

1,3,5-Trimethylbenzene (TMB, Mw 120.19 g/mol, AR, 97%), Tris (hydroxymethyl) aminomethane (TRIS, Mw 121.14 g/mol, ≥99.9%), dopamine hydrochloride (Mw 189.64 g/mol, 98%), Triamcinolone acetonide (TA), Lipopolysaccharides from *Escherichia coli* O55:B5 (LPS) and phenol were purchased from Aladdin (Shanghai, China). Pluronic^®^ F-127 (F127) was purchased from Sigma (Shanghai, China). *Bletilla striata* polysaccharide (BSP, Mw 390–460 kDa, ≥98%) was acquired from Xi’an Zelang Biotechnology (Xi’an, China). Sodium hyaluronic acid (HA, Mw 5–10 kDa) was obtained from Freda Biology Co., Ltd. (Shandong, China). The polydimethylsiloxane mold (PDMS, Shenzhen Thunder Cloud Information Technology Co., Ltd., China) had 225 (15 × 15) MNs, and the height and width of each needle were 700 and 300 μm, respectively. The distance among needles was 600 μm. TRITC-Phalloidin, 4′,6-diamidino-2-phenyllindole (DAPI) was purchased from Solarbio. Cell Counting Kit-8 (CCK-8) was purchased from Absin Bioscience Inc (Shanghai, China). Human Tumor Necrosis Factor Alpha (TNF-α) ELISA Kit was purchased from Elabscience Biotechnology Co., Ltd. (Wuhan, China). Methanol was the chromatographic grade for chromatographic analysis when High-performance liquid phase (HPLC) was used, and other chemical reagents were analytical grade, both provided by Sinopharm Chemical Reagent Co., Ltd. (Shanghai, China), and used directly.

### 2.2 Cell culture and animals

#### 2.2.1 Cell

In a humidified incubator (WIGGENS, Germany) at 37°C with 5% CO_2_, Human Oral Keratinocytes (HOK) and Human gingival fibroblasts (hGFs) were cultured using the complete medium. The complete medium includes 90% Dulbecco’s Modified Eagle Medium (DMEM, Biological Industries, Israel), 10% fetal bovine serum (FBS, Solarbio, Beijing, China) and 1% penicillin/streptomycin (Biological Industries, Israel).

#### 2.2.2 Animals

We purchased adult Sprague Dawley rats (SD, male, ∼150 g) from Jinan Pengyue laboratory animal breeding center (Shandong, China) and maintained them in Qingdao University’s Laboratory Animal Center in a specific pathogen-free (SPF) environment. All procedures performed in the study were in accordance with the Ethics Committee of Qingdao Stomatological Hospital Affiliated of Qingdao University Certificate (contract grant 2021KQYX031) and with the National Research Council’s Guide for the Care and Use of Laboratory Animals ethical standards.

### 2.3 Preparation of mesoporous polydopamine nanoparticles (MPDA NPs)

The preparation of MPDA NPs was carried out according to the methods reported in the previous literature (Yuan et al., 2020; Zeng et al., 2022). Briefly, 0.36 g of F127 and 417 μL of TMB solution were added to the mixture of 60 mL of ethanol and 65 mL of deionized water. After stirring on a magnetic stirrer for 30 min at room temperature, 90 mg of TRIS and 60 mg of dopamine hydrochloride were added to the mixture. The reaction was maintained for 24 h. The crude products were collected and washed at least three times with an ethanol/acetone mixture (v/v = 2: 1) to remove the template by centrifugation. Finally, MPDA NPs were obtained and dried in a vacuum drying oven (DZF-6050AB, China, Beijing).

### 2.4 Preparation of TA-loaded MPDA nanoparticles (TA@MPDA NPs)

Different concentrations of MPDA NPs suspension were prepared by dispersing MPDA NPs (5, 10, and 20 mg) uniformly in 10 mL of methanol under the action of sonication. TA solution (2 mg/mL, dissolved in methanol) was added dropwise to the MPDA NPs dispersion and stirred at 45°C for 3 h. Finally, TA@MPDA NPs were collected by centrifugation, dried by vacuum and stored at 4°C for further use. The amount of TA loaded into MPDA was calculated by subtracting the mass of TA in the supernatant from the total mass of the TA in the initial solution.

### 2.5 Characterization of MPDA and TA@MPDA NPs

The particle size distribution and surface charge of MPDA and TA-loaded MPDA were detected by dynamic light scattering (DLS, Zetasizer Nano ZS) at 25°C. All the tests were conducted in triplicate, and all the data were expressed as the mean ± standard (SD). The morphology of nanoparticles was observed by a JEM-2100 transmission electron microscopy (TEM, JEOL Ltd., Tokyo, Japan). The functional groups of MPDA and TA@MPDA in the range of 500–4,000 cm^−1^ were characterized using a Nicolet iN10 Fourier transform infrared (FTIR) spectrometer (Thermo Fisher Scientific, Waltham, MA, United States) performed 32 scans with a scan resolution of 2 cm^−1^. TA was determined by a Shimadzu LC-20A instrument (DGU-20A5R degasser, SIL-20A autosampler, CTO-20A column temperature chamber, SPD-M20A UV detector, LC-20AT infusion pump, and LabSolutions Chemical workstation, Kyoto, Japan) equipped with a SHIMADZU Shim-pack GIST C18 (250 × 4.6 mm, 5 μm) analytical column (P/N: 277-30017-08) using methanol-deionized water (65:35, v/v) as the mobile phases with a flow rate of 1.0 mL/min at 25°C. The regression equation with TA concentration as the independent variable and peak area as the dependent variable is Y = ×27681 − 27556 (*r* ^*2*^ = 0.9979) (Supplementary Figure S1A). The peak areas at 240 nm of the supernatants were collected. The drug loading (DL) and encapsulation efficiency (EE) of TA@MPDA were calculated via Eqs 1, 2, respectively:

$$DL=\frac{TA\;\text{loaded}\;\left(g\right)}{TA@MPDA\;\left(g\right)}\times 100\%$$ (1)

$$EE=\frac{Total\;TA\left(g\right)-free\;TA\;\left(g\right)}{Total\;TA\;\left(g\right)}\times 100\%$$ (2)

### 2.6 Fabrication and characterization of dissolvable microneedle patches

#### 2.6.1 Dissolving Microneedle Fabrication

0.1 mg MPDA or TA@MPDA was evenly dispersed in 1 mL deionized water by ultrasonic vibration. Then, HA and BSP were mixed in the ratio of 1:2, 1:1, 2:1, respectively, and dissolved in the above dispersion solution. The final concentration of HA was 30%. The microneedle molds injected with deionized water were placed in a vacuum at 0.08 mPa for 20 min so that each well was filled with deionized water, and the excess deionized water was removed. After that, the MPDA-HA/BSP or TA@MPDA-HA/BSP matrix solution was poured into the microneedle molds and then maintained 30 min under ultrasound until the cavity of the tips was filled with solution. The sample was then left at room temperature for approximately 48 h to dry completely. The corresponding MPDA-HA/BSP MNs or TA@MPDA-HA/BSP MNs were gently separated from the mold and stored in a dryer. In addition, HA/BSP MNs were prepared by the same method with HA/BSP as matrix solution.

#### 2.6.2 Morphology and mechanical property of MNs

The morphology of TA@MPDA-HA/BSP MNs was visualized by a dermoscope (AX-10, KaLanDe). In order to observe the microscopic structure of MNs, they were gilded by sputtering to increase conductivity after the edges of MNs were trimmed properly and then imaged using a scanning electron microscope (SEM, VEGA3, TESCAN, Czech Republic) at an accelerating voltage of 10 kV. The size of MNs was measured by ImageJ. The mechanical properties of MNs were tested by compression method at a temperature of 22°C ± 5°C and relative humidity of 40% ± 10%. TA@MPDA-HA/BSP MNs (array: 15 × 15) were pressed into the pig skin using thumb force. The insertion marks left were observed. In addition, TA@MPDA-HA/BSP MNs were inserted into the mucous membrane of rat tongue with the same force for 3 min, and hematoxylin and eosin (H&E) staining was performed to observe the depth of insertion.

#### 2.6.3 Dissolvability

The dissolution rates of TA@MPDA-HA/BSP MNs were studied on the tongue abdomen of SD rats. At the predetermined time points, MNs were removed from the tongue and observed by a microscope (OLYMPUS, Japan). The size variations of MNs were analyzed by ImageJ.

### 2.7 Preliminary evaluation of biosafety

#### 2.7.1 Blood compatibility

Citrated whole blood from healthy donors was diluted with normal saline (NS) to 2% (v/v). Then 50 μL of the diluted blood was added into the TA@MPDA-HA/BSP MNs dispersed NS solution with different concentrations (0.5, 1.0, 1.5, 2.0 mg/mL), and kept at 37°C for 1 h. Afterward, the mixtures were centrifuged at 1,500 rpm/min for 5 min. The absorbance of the supernatant was detected at 540 nm by a UV-Vis spectrophotometer (UV-8000, Shanghai Metash Instruments Co., Ltd., China). In addition, deionized water and NS solution were mixed with an equal volume of diluted blood to form the positive and negative control group, respectively. The hemolysis ratio was calculated as the following formula:

$$Hemolysis\;rate=\frac{A_{Sample}-A_{Negative\;control}}{A_{Positive\;control}-A_{Negative\;control}}\times 100\%$$ (3)

#### 2.7.2 Cytotoxicity

The cytotoxicity of MPDA, TA@MPDA, MPDA-HA/BSP MNs and TA@MPDA-HA/BSP MNs were evaluated using a standard CCK-8 assay. Human Oral Keratinocytes (HOK) were seeded into 96-well plates at a density of 3 × 10^3^ cells per well and incubated in at 37°C with 5% CO_2_ using the complete medium for 24 h. The cell culture medium was replaced by the test preparation diluted with culture medium for 24 h. After that, the medium was discarded, and the cells were cultured with 100 μL of CCK-8 reagent (10%) solution per well for 2 h. Subsequently, the optical density (OD) values were read with a microplate reader (800TS, Bio-Tek, China) at a wavelength of 450 nm. Pristine culture medium was used as a control group. Cell viability was calculated according to the formula:

$$Cell\;viability=\frac{OD_{Preparation}-OD_{Blank}}{OD_{Control}-OD_{Blank}}\times 100\%$$ (4)

where OD_Preparation_, OD_Control_, and OD_Blank_ refer to the OD values of the test preparation-treated group, pristine medium-treated group, and the culture medium without cells, respectively. Each group was carried out in three replicates.

### 2.8 Cell Morphology Staining

TRITC-Phalloidin and DAPI staining were used to detect the effect of the MN patches on cell morphology. Human gingival fibroblasts (hGFs) were inoculated into 24-well plates at a density of 5.0 × 10^4^ cells per well. After incubation for 24 h, the original medium of experimental group was replaced by a MN-contained medium (MPDA-HA/BSP MNs or TA@MPDA-HA/BSP MNs, 2 mg/mL) and the control group was replaced by the complete medium. Cells were fixed with 4% (v/v) paraformaldehyde to maintain morphology, and treated with 0.5% (w/v) Triton X-100 for 3 min and 5% bovine serum albumin for 30 min, with phosphate buffered saline (PBS) used for cleaning. Then, cells were stained with TRITC-Phalloidin and DAPI under dark conditions. The morphology of the cells was photographed using a fluorescence microscope (OLYMPUS, Japan).

### 2.9 Cell scratching assay

The cell scratching assay was performed using hGFs. Briefly, hGFs were seeded on 6 well culture-treated plates with a density of 5 × 10^5^ cells each well and cultured for 24 h. To verify that cell migration was caused by drug action rather than cell proliferation, cells were maintained in a serum-free starvation state for 24 h to minimize proliferation. Then, three straight lines were drawn evenly using a 10 μL tip for the formation of microdamage and the fallen cells were washed and removed with PBS, after which the microneedle-contained medium (2 mg/mL) was then introduced into each well. Wound closure was monitored and imaged using an optical microscope (OLYMPUS, Japan) in 12 h intervals. ImageJ was used to quantify the wound area. The formula is as follows:

$$Degree\;of\;wound\;closure=\frac{S_{0}-S_{A}}{S_{0}}\times 100\%$$ (5)

where S_0_ refers to the initial wound area (0 h), and S_A_ refers to wound area of 12 and 24 h. Each group was carried out in three replicates.

### 2.10 Anti-inflammatory test

To verify the anti-inflammatory ability of the prepared microneedle patches, LPS-induced hGFs were used to construct a cell inflammation model. hGFs were inoculated into 96-well plates at a density of 3 × 10^3^ per well for 24 h. After removing the original medium, the cells were co-cultured with LPS and MN patches for 24 h. Only adding medium was used as blank control group. LPS and culture medium were used as the positive control group. The final concentrations of LPS and MN patches were 0.1 and 2 mg/mL, respectively. The level of TNF-α in the supernatant was determined by enzyme-linked immunosorbent assay (*n* = 3).

### 2.11 Insertion safety of MN patches

To evaluate the safety of TA@MPDA-HA/BSP MNs insertion, the MNs were inserted into the tongue mucosa of rats using thumb force to observe the recovery of the mucosa. After 3 min, the MNs were completely dissolved, and then were removed. Before the insertion of the MNs, immediately and 5, 10, and 30 min after the insertion, photos of the tongue mucosa were recorded. Twenty-4 hours later, the rats were euthanized, and the tongue tissue was removed for pathological section preparation and H&E staining.

### 2.12 Study of the therapeutic effect of MN patches for ulcer model *in vivo*

The model of oral ulcer was constructed by chemical burning as our previous studies (Zhu et al., 2022). SD rats were anesthetized by intraperitoneal injection of sodium pentobarbital (50.0 mg/kg). A cotton ball immersed in 90% phenol solution was placed at one end of a glass tube and exposed to the mucous membrane of rat tongue abdomen for 60 s. The oral ulcer model was successfully established. On the first day after modeling, the rats were randomly divided into five groups: control, triamcinolone acetonide dental paste (Ning Zhi Zhu^®^) group, HA/BSP MNs group, MPDA-HA/BSP MNs group, TA@MPDA-HA/BSP MNs group. Three rats were assigned for each group, and the healing of the ulcer was recorded using a digital camera for analysis. On the fifth day after administration, the rats were euthanized, and tongue tissue was excised. H&E staining and Masson’s Trichrome staining were used to detect oral ulcer healing degree. The levels of TNF-α and CD31 in each group were observed by immunohistochemistry (IHC) to evaluate the ulcer repair.

### 2.13 Statistical analysis

All quantified data were presented as mean ± standard deviation (SD). All statistical analysis graphs were drawn using GraphPad Prism 9 or Origin 2021. One-way analysis of variance (ANOVA) with Tukey`s test was used to determine the differences between groups. A value of *p* < 0.05 was considered to be statistically significant.

## 3 Results and discussion

### 3.1 Preparation and characterization of MPDA and TA@MPDA NPs

**FIGURE 2:**
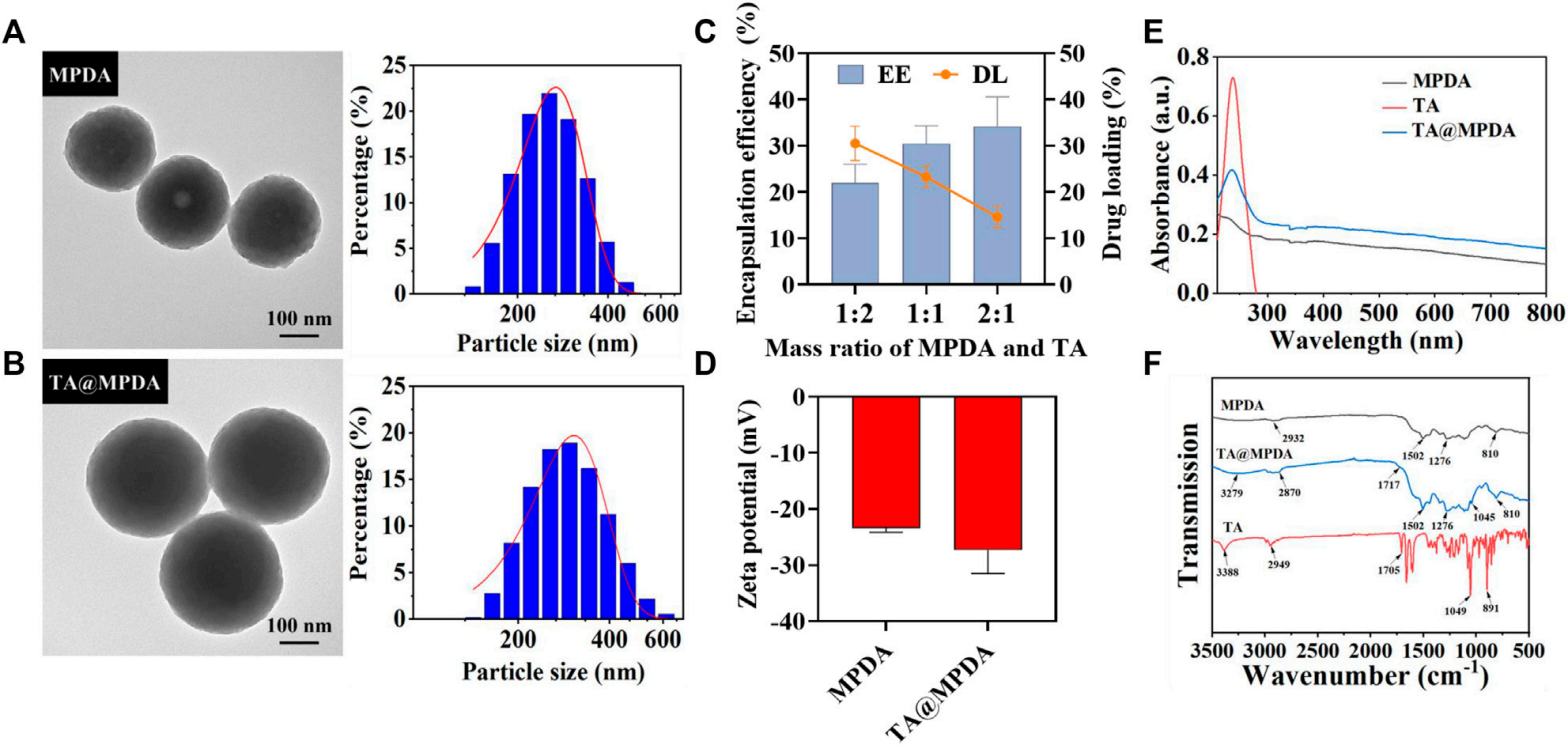
Characterization of **(A)** MPDA and **(B)** TA@MPDA TEM Images and particle size distributions. **(C)** The EE (%) and DL (%) of TA and MPDA in different proportions. **(D)** Zeta potential of TA, MPDA and TA@MPDA, respectively. **(E)** UV–Vis absorption spectra of TA, MPDA, and TA@MPDA, respectively. **(F)** The FTIR spectra of TA, MPDA, and TA@MPDA.

MPDA NPs were synthesized by oxidative self-polymerization and self-assembly of dopamine under alkaline conditions (Yuan et al., 2020). The typical structure of MPDA NPs was observed from transmission electron microscopy (TEM), and their surface had obvious mesoporous structure with an average particle size of 241.97 ± 7.54 nm and a narrow size distribution (Figure 2A). Then, triamcinolone acetonide (TA) was loaded on the surface of MPDA. As shown in Figure 2B, the morphology of TA@MPDA tended to be flat after loading TA and the size of NPs increased to 297.16 ± 8.12 nm. The particle size of nanoparticles subjected to drug loading increased. With decrease of TA/MPDA mass ratio in feed, the drug loading decrease, whereas, the encapsulation efficiency and stability increased according. In consideration of drug loading and encapsulation efficiency, 1:1 was selected for further applications and was characterized in detail. The high-performance liquid chromatography was shown in Supplementary Figure S1B. The DL and EE were 23.27% ± 1.91% and 30.43% ± 3.18%, respectively (Figure 2C). The zeta potential of TA@MPDA NPs decreased from −23.37 ± 0.63 mV to −27.30 ± 3.42 mV (Figure 2D). It was demonstrated that the negatively charged NPs had good colloid stability and can be evenly distributed in MNs (Cheng et al., 2020). In the UV-Vis spectrum of TA@MPDA, the new characteristic absorption peak of triamcinolone acetonide shown at ∼ 237 nm (λ _Max_) demonstrated that the successful loading of TA into MPDA NPs (Figure 2E). To investigate the intermolecular interaction between MPDA and TA, FTIR detection was performed. The characteristic peaks of TA skeleton appear at 3,388, 2,949, 1,075, 1,049, and 891 cm^−1^, which is corresponding to–OH, C–H, C=O and C–C tensile vibration, respectively. The MPDA skeleton has characteristic peaks at 2,932 and 810 cm^−1^, which is C–H and C–C tensile vibration, respectively. The characteristic peaks of TA@MPDA skeleton at 3,279, 2,870, 1,717, 1,045 and 810 cm^−1^ were the tensile vibration of–OH, C–H, C=O, respectively. In addition, both the MPDA and TA@MPDA skeletons have the characteristic peaks at 1,502 and 1,276 cm^−1^ (Figure 2F). The above experiments proved that TA was successfully loaded.

### 3.2 Preparation and characterization of the dissolving microneedle patch

**FIGURE 3:**
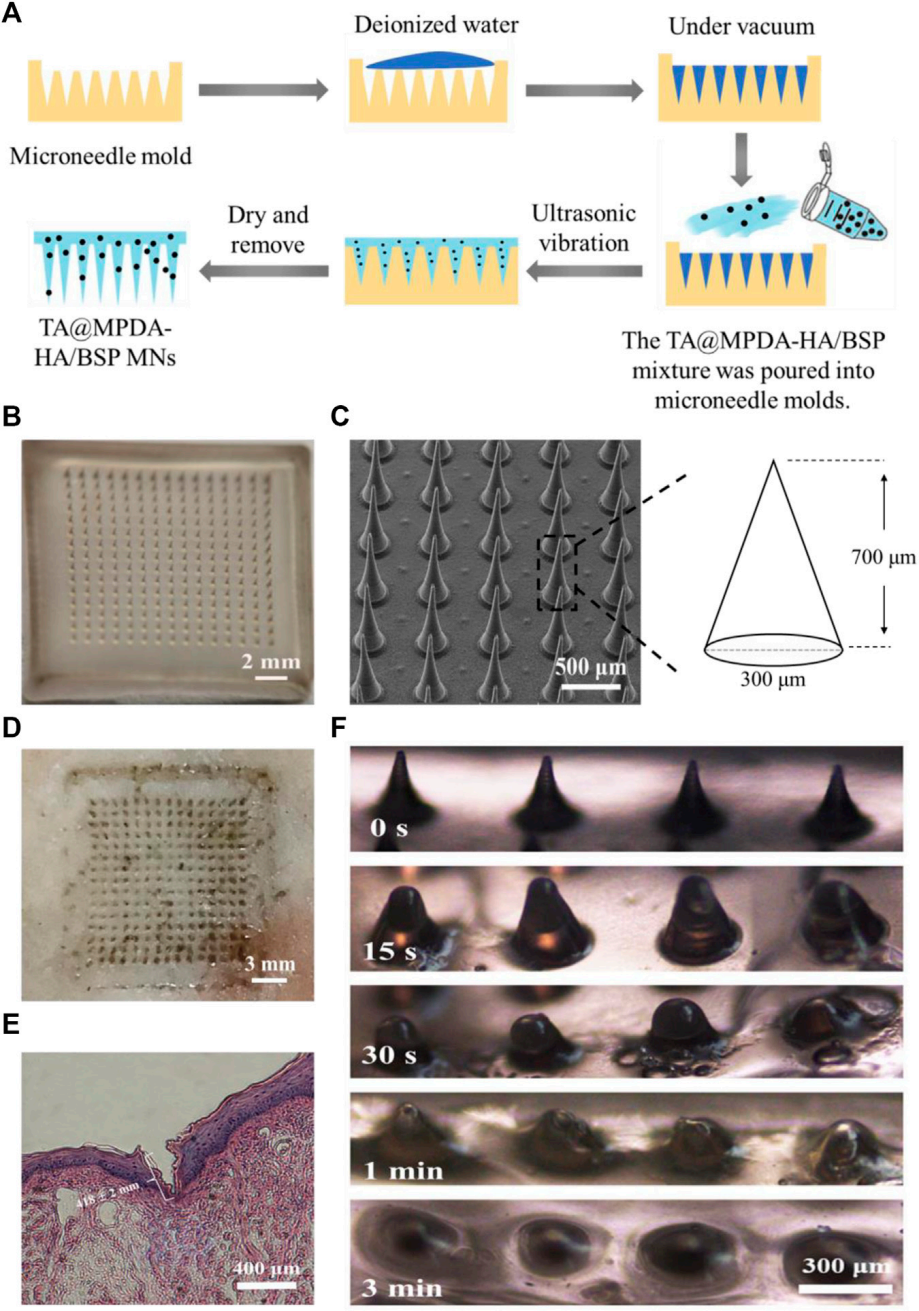
Characterization of MN patch. **(A)** One-casting fabrication process of the microneedles. **(B)** Images of TA@MPDA-HA/BSP MNs taken using a microscope. **(C)** SEM image of TA@MPDA-HA/BSP MNs. **(D)** Photo of pig skin after microneedle insertion (15 × 15). **(E)** The image of H&E staining image of the rat tongue mucosa after being treated with MN patch. **(F)** Solubility of TA@MPDA-HA/BSP MN patch inserted into oral mucosa at different times.

As shown in Figure 3A, the microneedles were fabricated by a one-casting fabrication process. The results showed that when the ratio of HA to BSP was 1:2 or 1:1, the demolding time of MNs was longer and the tip was difficult to form. When the ratio was 2:1, the MNs can be completely dried within 2 days and successfully demolded. In the dermoscopy (Figure 3B) and SEM (Figure 3C) images, the microneedle patch consisted of 15 × 15 arrays, which showed a well-arranged regular cone without bubbles and needle breakage. In the process of SEM, a certain Angle was set for the MNs to achieve better imaging, so the size of the MNs presented in the SEM image was smaller than the actual size. After pressing the MNs that have been loaded with TA@MPDA into a pig skin for 3 min, complete dark arrays (15 × 15) were left on the surface of the skin, indicating that the MNs had enough mechanical strength (Figure 3D). The histological section of the MN penetrated oral mucosa also showed a clear shape of microneedle penetration and the insertion depth was 418 ± 2 mm (Figure 3E). The solubility of MNs after puncture of oral mucosa directly affected the release and distribution of their payload. As shown in Figure 3F, the sharp needle of microneedles completely dissolved within 3 min after insertion into the lingual mucosa of rats. The above results manifested that the mechanical strength of the microneedles was sufficient to penetrate and transport the cargo to the interior of the wound site.

### 3.3 Preliminary evaluation of biosafety

#### 3.3.1 Blood compatibility

**FIGURE 4:**
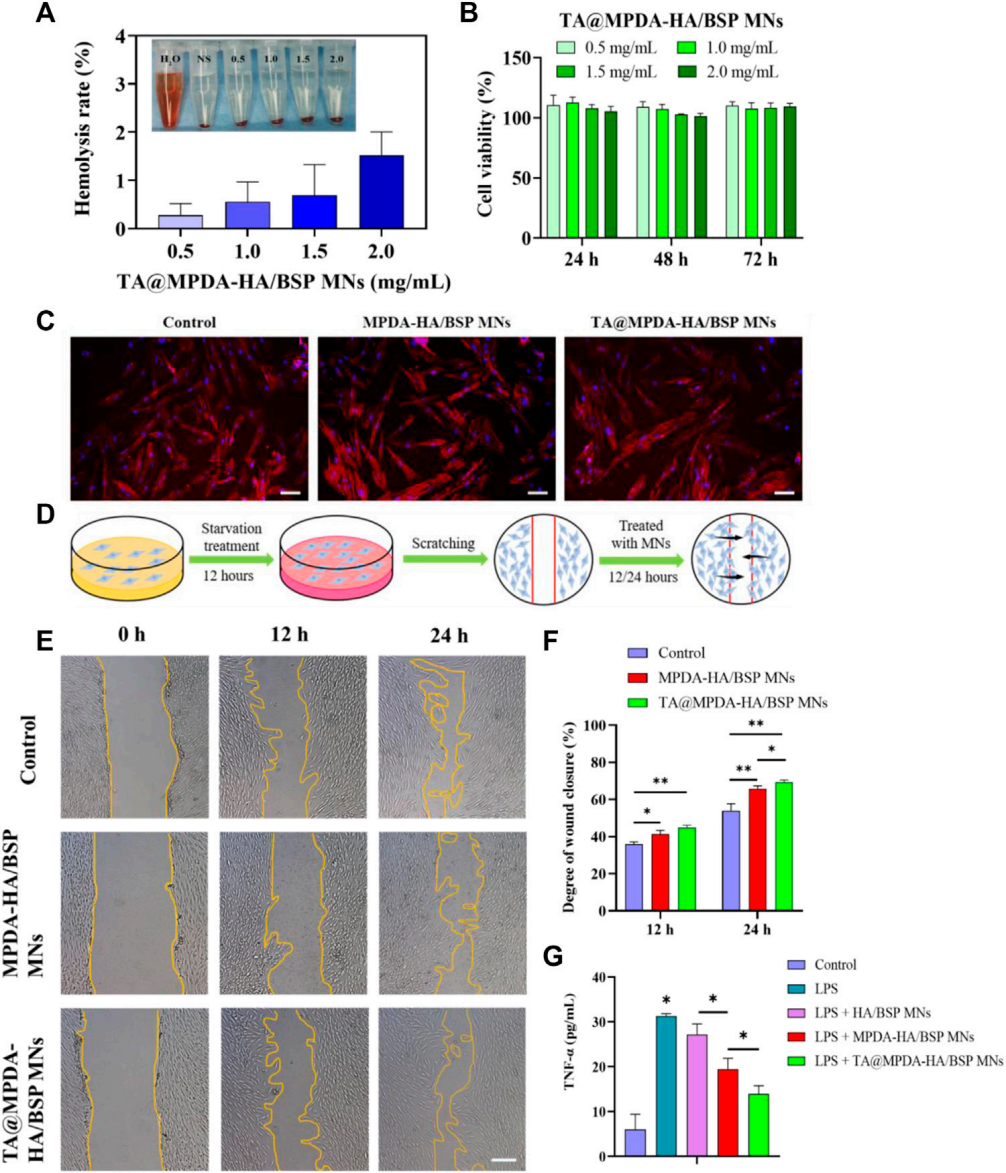
**(A)** Hemolysis ratio of whole blood by TA@MPDA-HA/BSP MNs (0.5, 1.0, 1.5, 2 mg/mL). **(B)** Cell viability of HOK after the incubation with TA@MPDA-HA/BSP MNs. **(C)** Fluorescent images of hGFs treated with blank, MPDA-HA/BSP MNs and TA@MPDA-HA/BSP MNs. The red staining is actin cytoskeleton and the blue staining is the cell nucleus (scale bars: 50 μm). **(D)** Schematic diagram of a cell scratch experiment. **(E)** Typical images show hGFs wound closure treated with blank, MPDA-HA/BSP MNs and TA@MPDA-HA/BSP MNs at 12 and 24 h (scale bars: 200 μm). **(F)** Degree of wound closure in each group of the scratch test. **(G)** To test the anti-inflammatory ability of the MN patches, levels of TNF-α were analyzed by ELISA.

The hemocompatibility of microneedles is one of the key factors for the overall success of clinical applications (Zhao et al., 2017; Wu et al., 2021). As described above, we performed hemolysis assays to elucidate their blood compatibility of TA@MPDA-HA/BSP MNs to whole blood in biological solutions. The illustration in Figure 4A exhibited that the color of the supernatant of the MN groups was similar to that of the negative control group, which was almost colorless, while the distilled water group was distinctly red. According to the authoritative evaluation standard of ASTM F756-17, if the clinic biomedical materials with a hemolysis rate are less than 2%, it can be considered admissible. Quantification demonstrated that the hemolysis ratio of different concentrations (0.5–2.0 mg/mL) was <2%, no hemolysis occurred (Figure 4A). These data indicated that TA@MPDA-HA/BSP MNs have good blood compatibility.

#### 3.3.2 Cytotoxicity

The cytotoxicity of both MPDA and TA@MPDA groups gradually increased as the drug concentration increased in the range of 25–200 μg/mL, showing concentration dependence (Supplementary Figure S2A, B). The cell viability of 25–100 μg/mL MPDA and TA@MPDA were above 58.55% ± 1.58% and 50.84% ± 1.99%, respectively. There were significant differences (*p* < 0.05) in cell viability between 100 μg/mL and 200 μg/mL of the two kinds of particles. The statistical results showed that there was a statistical difference between 0.5 and 2 mg/mL MPDA-HA/BSP MNs groups (Supplementary Figure S2C) at 24 h (*p* < 0.05) and there were no statistical differences between TA@MPDA-HA/BSP MNs groups (*p* > 0.05) (Figure 4B). Two kinds of MNs had cell viability above 101.55% ± 1.77%. These results indicated that HA and BSP may have the effect of promoting cell proliferation. The above experiments proved that the drug-loaded MNs had good biocompatibility, providing a reliable guarantee for use as oral mucosal patches.

#### 3.3.3 Cell Morphology Staining

The cytoskeleton was stained with TRITC-Phalloidin and DAPI to observe the effect of microtargeting on cell morphology. After staining, the cell membrane of hGFs showed red spindle shapes, and the cell nucleus was filled, and the nucleus showed blue round shapes, which corresponded to the cell membrane one by one (Figure 4C). The results showed that the morphology of hGFs in the experimental group did not change significantly compared with the control group.

### 3.4 Cell scratching assay

The process of wound-scratch testing was shown in Figure 4D. Upon quantification, the MPDA-HA/BSP, TA@MPDA-HA/BSP MNs and the control group displayed scratch closures of 65.83% ± 1.24%, 69.40% ± 0.93%, and 54.01% ± 3.05% after 24 h, respectively (*p* < 0.05, Figure 4F). These data showed that the migration rate of hGFs treated with MPDA-HA/BSP MNs solution was significantly faster than that of the control group, and the migration rate of hGFs treated with TA@MPDA-HA/BSP solution was the fastest (Figure 4E). These results indicated that the MPDA-HA/BSP, TA@MPDA-HA/BSP MNs had the ability to promote wound repair and thus promote the healing of oral ulcer.

### 3.5 Anti-inflammatory test

To our knowledge, MPDA and Chinese herbs extract BSP have been widely explored in the treatment of inflammatory diseases (Zhang et al., 2010; Chen et al., 2021). To verify the synergistic anti-inflammatory effect of TA@MPDA-HA/BSP MNs, we investigated tumor necrosis factor (TNF-α) levels in the supernatant of hGFs after induction by lipopolysaccharides (LPS). As shown in Figure 4G, the TNF-α level in MPDA-HA/BSP MNs group was significantly lower than that in HA/BSP MNs group (*p* < 0.05), and remarkably, the TA@MPDA-HA/BSP MNs group exhibited the lowest TNF-α concentration. From these data we conclude that the superimposition of multiple components of the microneedles enhance their anti-inflammatory effect, which may achieve the goal of low-dose hormones for suppression of inflammation.

### 3.6 Insertion safety of MN patches

The safety of TA@MPDA-HA/BSP MNs was evaluated by inserting MNs into the tongue mucosa of rats and observing the inflammatory response of the mucosa. Normal SD rats tongue mucosa were selected for the experiment. As was shown in Supplementary Figures S3A–E, immediately after insertion, the pores left by the needle array can be seen in the lingual mucosa. After 5 min, the pore scope was significantly reduced. After 10 min, the pores basically disappeared. 30 min after insertion, the lingual mucosa returned to the normal state before insertion. No obvious hyperemia was observed in the mucosa during the whole process. H&E staining was performed on tongue tissue 24 h after MN application. The results showed that the pathological images of the TA@MPDA-HA/BSP MNs group and the control group were basically the same, and no obvious inflammatory cell infiltration was observed (Supplementary Figures S3E, F). The above phenomenon proved that MNs have good insertion safety, which provides an important basis for clinical application.

### 3.7 *In vivo* animal experiments

**FIGURE 5:**
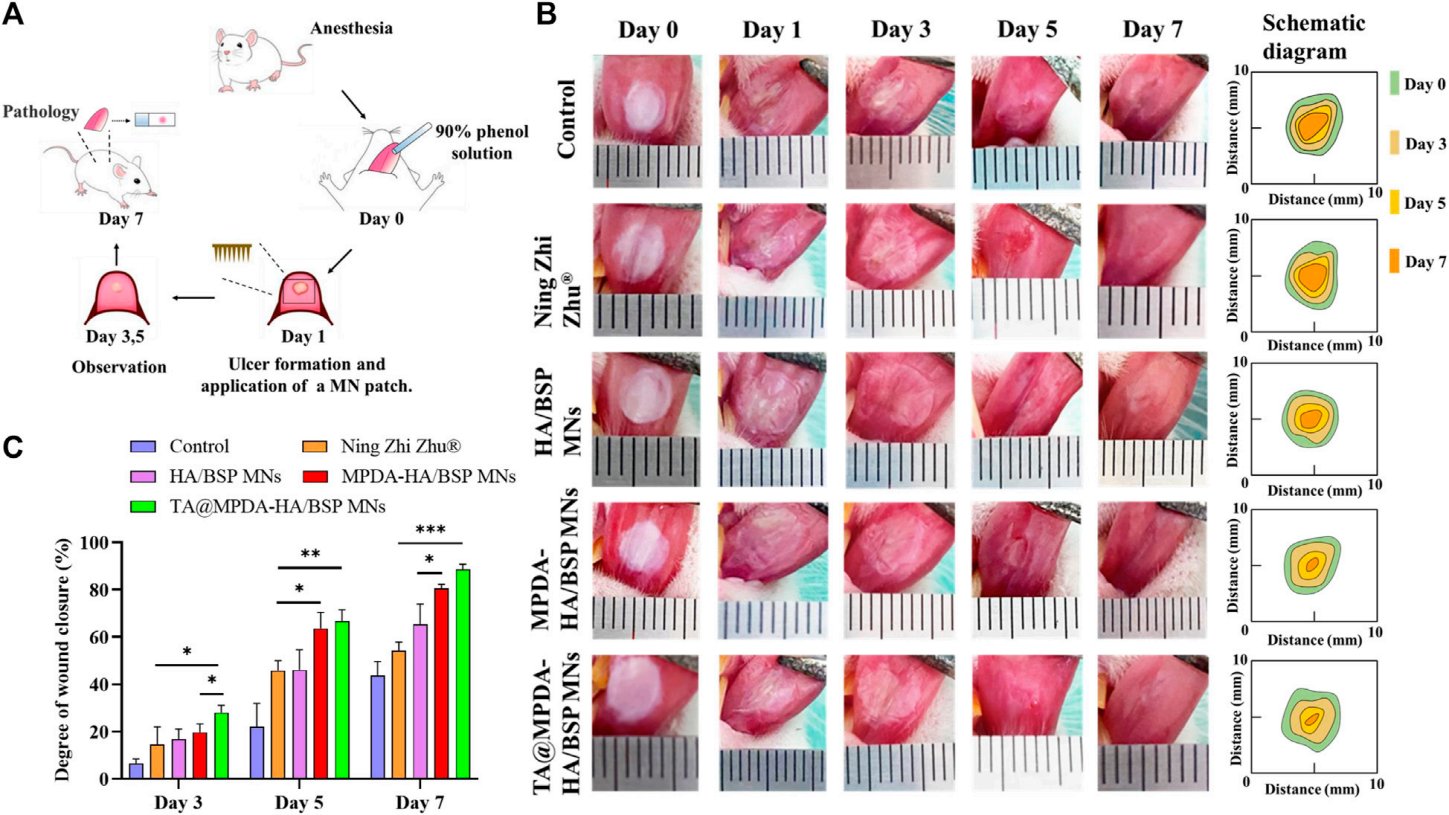
**(A)** A schematic modeling and treatment process of rat oral ulcer. **(B)** Photos and diagrammatic drawing of oral ulcer changes in each group on the day of ulcer formation, before treatment, and on day three, five, and seven after treatment. **(C)** Degree of wound closure at day three, five, and seven of each group.

In this study, the ability of MNs to promote oral ulcer wound healing was evaluated by establishing and treating oral ulcer models in SD rats (Figure 5A). The photographic images of the oral ulcer modeling and after treatment with Ning Zhi Zhu^®^ and microneedle samples were shown in Figure 5B. On the first day, oval or round ulcers with a diameter of approximately 5 mm were formed. The established ulcer models appeared to be hollow in the center, covered with a yellow and white pseudomembrane, and congested and swollen at the edges. Through quantization, as shown in Figure 5C, the TA@MPDA-HA/BSP MNs and MPDA-HA/BSP MNs treated wounds closed faster from the third day compared to Ning Zhi Zhu^®^ and nontreated groups (*p* < 0.05). Moreover, the MPDA-HA/BSP MNs group showed better therapeutic effects than the HA/BSP MNs (*p* < 0.05), suggesting that MPDA NPs are effective in treating oral ulcers (Fu et al., 2021; Zhang et al., 2021). On day seven, the degree of wound closure was 43.59% in the control group, 54.31% in the Ning Zhi Zhu^®^ group, 65.27% in the HA/BSP MNs group, 80.60% in the MPDA-HA/BSP MNs group, and 88.57% in the TA@MPDA-HA/BSP MNs group. Remarkably, the dose of TA in the TA@MPDA-HA/BSP MNs group was only 10% of that in the clinic ointment Ning Zhi Zhu^®^, however, its therapeutic effect was equal to or better than the latter, which realized the original intention of reducing the dosage of glucocorticoid hormone. In addition, due to the anesthetic effect, the rats could not chew for a certain period of time, increasing the residence time of Ning Zhi Zhu^®^ on the mucosa. However, when the rats were in normal activity, the mastication was more frequent, which made it more difficult for the ointment to stay on the mucous and take effect. The microneedle drug delivery system, on the other hand, could break the mucosal barrier and deliver the drug quickly and give full effect due to its transmucosal delivery ability and adsorption capacity.

**FIGURE 6:**
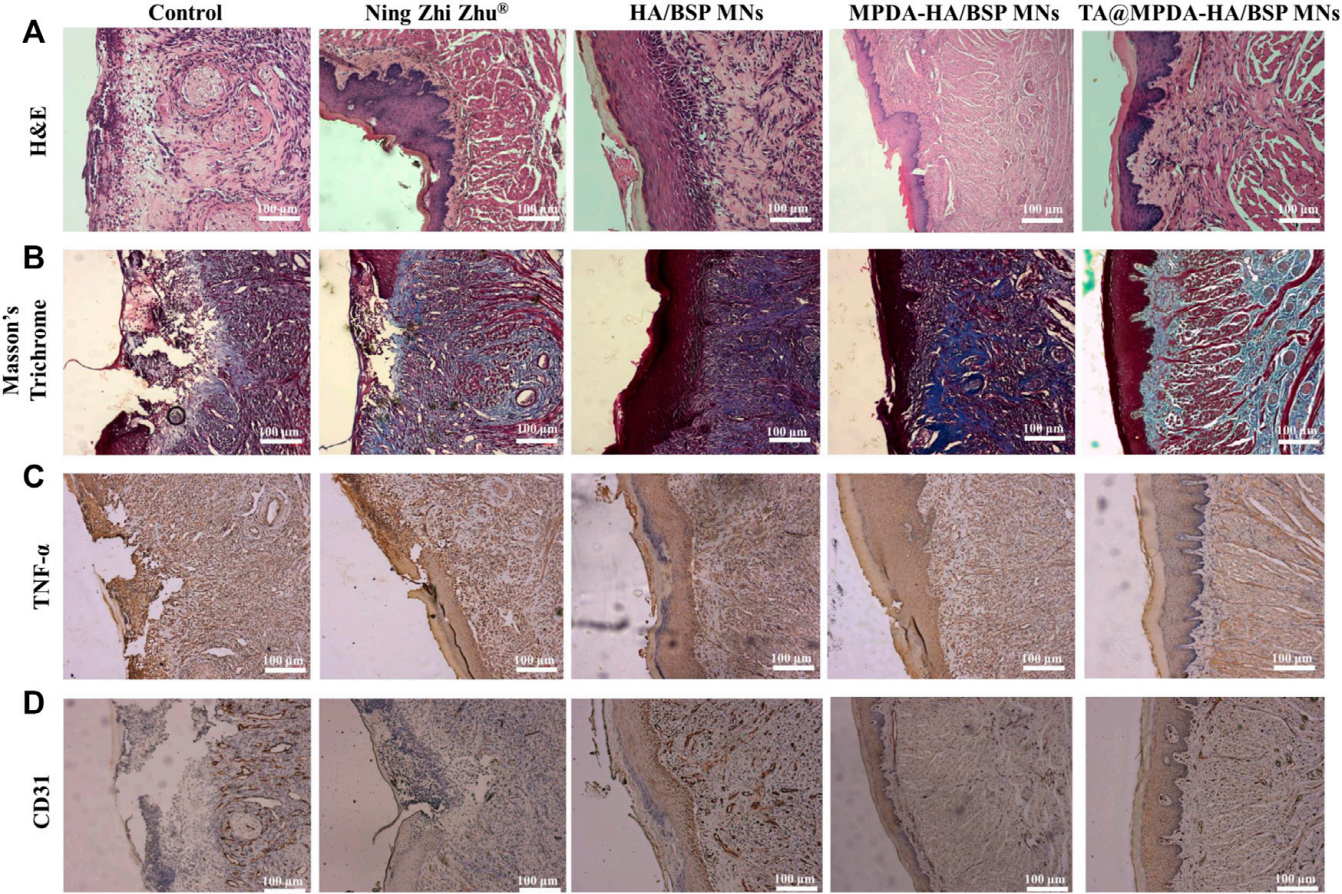
**(A)** H&E staining and **(B)** Masson’s Trichrome staining of rat tongue ulcers in each group after treatment. **(C)** TNF-α and **(D)** CD31 IHC after treatment.

To further detect the degree of ulcer healing, H&E staining (Figure 6A) and Masson’s Trichrome staining (Figure 6B) had been used to evaluate the quality of oral regenerated tissue in each group. In the control group, a large number of inflammatory cells were infiltrated in the submucosa, including neutrophils and plasma cells, and the upper cortex was shed. The damaged area was in direct contact with the fibrin layer, and the healing was slow. The epithelial tissues of Ning Zhi Zhu^®^ group and HA/BSP MNs group were discontinuous, with inflammatory cells in the submucosa, thickened basement membrane and irregular connective tissue hyperplasia. In the MPDA-HA/BSP MNs group, the stratum corneum was still missing, epithelial cells were edematous, and new connective tissue formed under the basement membrane. The epithelium of TA@MPDA-HA/BSP MNs group was basically intact without obvious inflammatory cells. By observing the blue trend of Masson’s Trichrome staining, it can be seen that MPDA-HA/BSP MNs group and TA@MPDA-HA/BSP MNs group both showed good collagen regeneration. Collagen production plays an important role in oral mucosal tissue repair (Wu et al., 2016). TNF-α is a pro-inflammatory cytokine produced primarily by macrophages and monocytes, and is involved in normal inflammatory and immune responses. The results showed that the TA@MPDA-HA/BSP MNs group had significantly lower levels of inflammation than the other groups (Figure 6C). CD31 may be involved in leukocyte migration, angiogenesis and integrin activation. As can be seen from Figure 6D, the number of new blood vessels in the TA@MPDA-HA/BSP MNs group was the highest. Experimental results suggest that TA@MPDA-HA/BSP MNs may accelerate oral ulcer healing by promoting collagen and neovascularization and reducing inflammation levels in tissues.

## 4 Conclusion

A HA/BSP-based dissolving microneedle was developed to mediate mesoporous polydopamine nanoparticles (MPDA) loaded with Triamcinolone acetonide (TA) for the treatment of oral mucositis (OM). TA can be successfully loaded into MPDA, thus improving bioavailability of TA. TA@MPDA-HA/BSP MNs had well-arranged microneedles array, high penetration efficiency, rapid dissolution in 3 min, excellent biocompatibility and anti-inflammatory properties. When TA@MPDA-HA/BSP MNs were used to treat oral ulcers, the area of oral ulcers and inflammatory factor level such as TNF-α and CD31 were significantly reduced. TA@MPDA-HA/BSP MNs can not only improve the bioavailability of TA, but also play the role of MPDA, BSP and HA in the combination treatment of OM, becoming a new clinical comfortable and effective alternative for the treatment of OM.

## Data Availability

The original contributions presented in the study are included in the article/Supplementary Material, further inquiries can be directed to the corresponding authors.
