# Supplementary material for: Microneedle patches containing mesoporous polydopamine nanoparticles loaded with triamcinolone acetonide for the treatment of oral mucositis
Source: Front Bioeng Biotechnol. 2023 May 5;11:1203709. doi: 10.3389/fbioe.2023.1203709 (PMC10196213; doi:10.3389/fbioe.2023.1203709)
Supplement: Supplementary file 1 [file DataSheet1.docx]

Supplementary Material

Microneedle patches containing mesoporous polydopamine nanoparticles loaded with triamcinolone acetonide for the treatment of oral mucositis

Xiaoying Qu, Xiaoli Guo, Tingting Zhu, Zhe Zhang, Wanchun Wang*, Yuanping Hao*

*** Correspondence:** Yuanping Hao: yphao@qdu.edu.cn (Y.H.); Wanchun Wang: wangwanchun2019@qdu.edu.cn (W.W.)


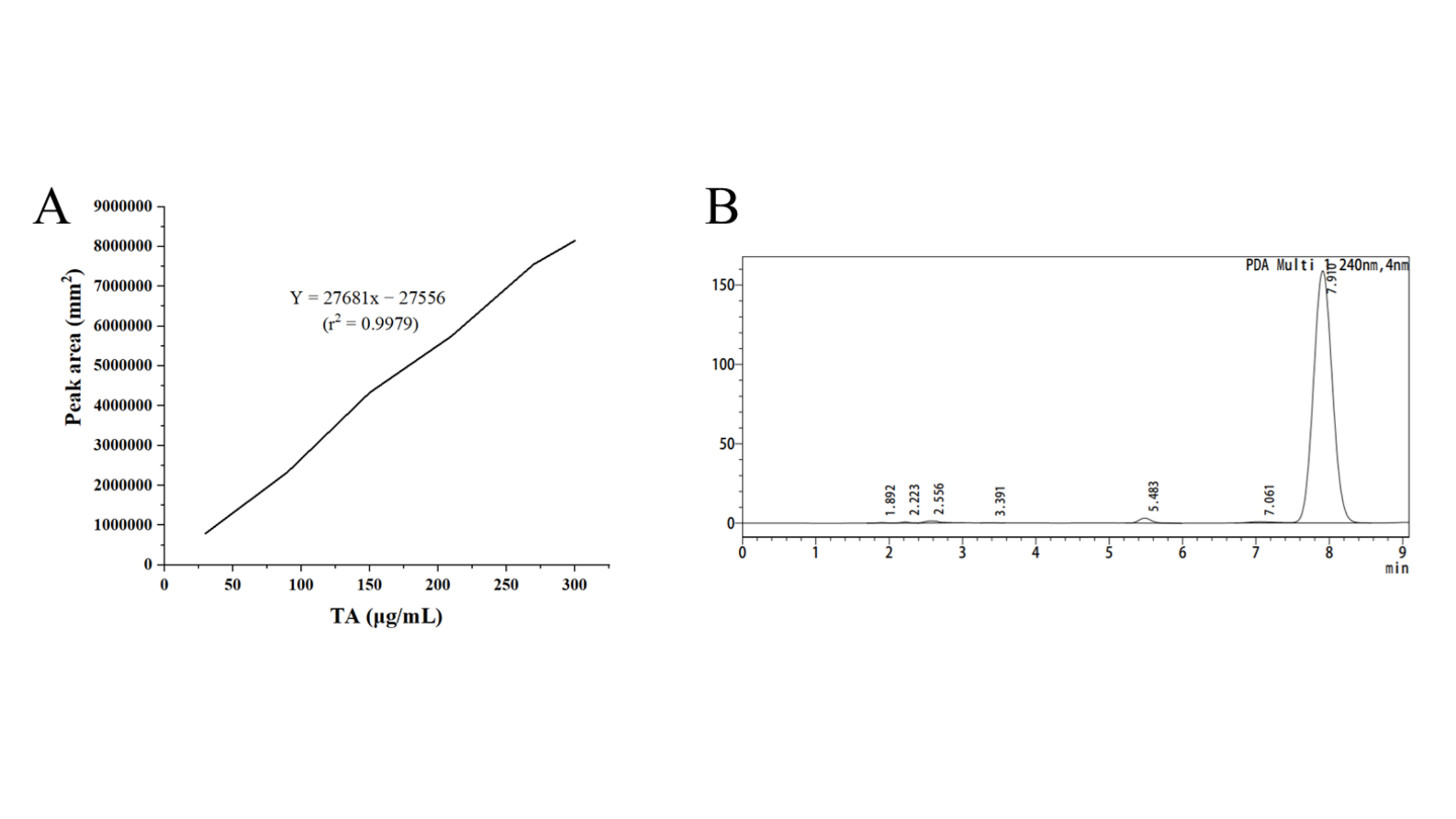


**FIGURE S1**

**(A)** The regression equation with TA concentration as the independent variable and peak area as the dependent variable is Y = 27681x − 27556 (r^2^ = 0.9979). **(B)** High performance liquid chromatography of 1:1 (mass ratio of MPDA: TA) supernatant.

***
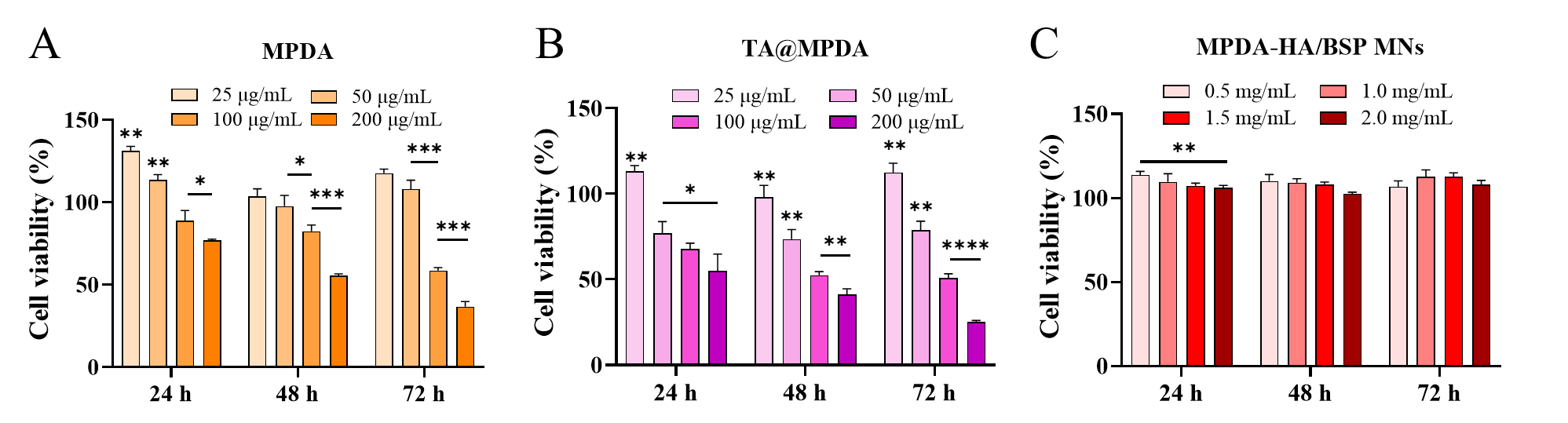
***

**FIGURE S2**

Cell viability of HOK after the incubation with **(A)** MPDA, **(B)** TA@MPDA, **(C)** MPDA-HA/BSP MNs.

***
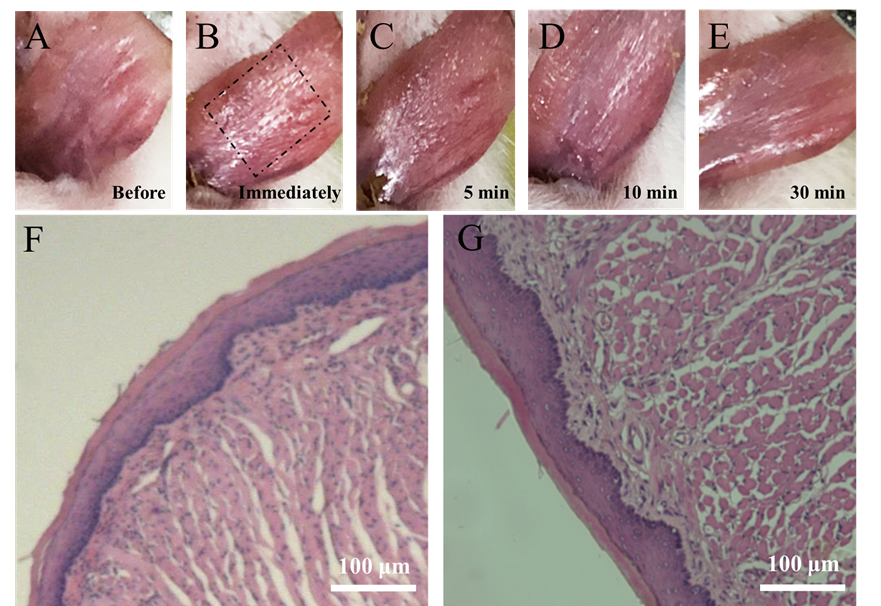
***

**FIGURE S3**

Photos of the tongue mucosa **(A)** before, **(B)** immediately, **(C)** 5, **(D)** 10, and **(E)** 30 minutes after the TA@MPDA-HA/BSP MN insertion. H&E staining images of **(F)** the tongue mucosa after microneedle insertion for 24 hours and **(G)** the normal tongue mucosa.
